# Marine Origin Polysaccharides in Drug Delivery Systems

**DOI:** 10.3390/md14020034

**Published:** 2016-02-05

**Authors:** Matias J. Cardoso, Rui R. Costa, João F. Mano

**Affiliations:** 1 3B’s Research Group—Biomaterials, Biodegradables and Biomimetics, University of Minho, Headquarters of the European Institute of Excellence of Tissue Engineering and Regenerative Medicine, Avepark—Parque de Ciência e Tecnologia, Zona Industrial da Gandra, 4805-017 Barco GMR, Portugal; matjcardoso@gmail.com; 2 ICVS/3B’s, PT Government Associated Laboratory, Braga/Guimarães, Portugal

**Keywords:** drug delivery, polysaccharides, marine excipients, biomaterials, polysaccharide/drug conjugates

## Abstract

Oceans are a vast source of natural substances. In them, we find various compounds with wide biotechnological and biomedical applicabilities. The exploitation of the sea as a renewable source of biocompounds can have a positive impact on the development of new systems and devices for biomedical applications. Marine polysaccharides are among the most abundant materials in the seas, which contributes to a decrease of the extraction costs, besides their solubility behavior in aqueous solvents and extraction media, and their interaction with other biocompounds. Polysaccharides such as alginate, carrageenan and fucoidan can be extracted from algae, whereas chitosan and hyaluronan can be obtained from animal sources. Most marine polysaccharides have important biological properties such as biocompatibility, biodegradability, and anti-inflammatory activity, as well as adhesive and antimicrobial actions. Moreover, they can be modified in order to allow processing them into various shapes and sizes and may exhibit response dependence to external stimuli, such as pH and temperature. Due to these properties, these biomaterials have been studied as raw material for the construction of carrier devices for drugs, including particles, capsules and hydrogels. The devices are designed to achieve a controlled release of therapeutic agents in an attempt to fight against serious diseases, and to be used in advanced therapies, such as gene delivery or regenerative medicine.

## 1. Introduction

Marine organisms are a vast source of different compounds with diverse biological properties and bioactivity. Recently, a growing interest in many scientific areas that study the diverse applications of marine compounds has been found, justified by their large biodiversity and simplicity of the extraction and purification processes [1,2]. Marine biomaterials have wide applicability in biomedicine because of their noncytotoxic characteristics, biodegradability and biocompatibility. These biological properties have allowed the discovery of a broad range of novel bioactive compounds with pharmacological properties and constitute a fundamental cornerstone of the pharmaceutical industry [2,3,4]. Some of these compounds have been studied for cancer treatment due to their antitumoral properties [5,6,7], among which are polypeptides extracted from tunicates [8] and sponges [9]. Many of these compounds are already used clinical trials, such as Aplidin [10] and Ecteinascidin 743 [11].

Marine polysaccharides are mostly used in food and cosmetic industries, but are also widely present in pharmaceutical sciences, with an increasing interest in integrating them as materials for the incorporation of bioactive agents [12]. Marine algae are the main source of marine polysaccharides, but they can also be obtained from animal sources, such as the skeletons of crustaceans and cartilaginous fish tissue. There are also some polysaccharides that can be extracted from marine microorganism, like some prokaryotes [13]. Marine polysaccharides can be described as a large complex group consisting of different macromolecules with different biological properties [14,15]. Polysaccharides may exhibit different chemical structures and different biological properties such as biocompatibility, biodegradability, adhesive properties and the ability to form hydrogels. Among the many marine polysaccharides there is one group that stands out: sulfated polysaccharides [16]. In comparison with other marine polysaccharides, they exhibit bioactivities that include antioxidant [17], anticoagulant [18], anticancer [19], antiviral [20], anti-allergic [21], anti-adhesive, anti-angiogenic and anti-inflammatory actions [22]. The systematic study of some of these materials for drug delivery systems (DDSs) allowed discovering new chemical modification methods aiming to harness such biological activities and change their affinity to specific drugs. Considering the latter, it has been possible to increase the ability to incorporate drugs and increase the efficacy of their release, either by chemical reactions or by interactions with other natural or synthetic polymers [23].

**Figure 1:**
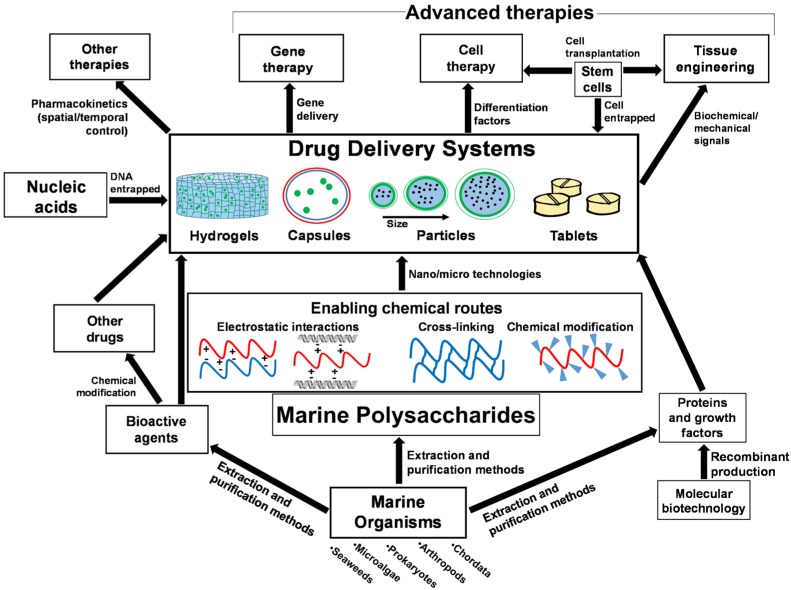
Interrelations of marine origin polysaccharides in drug delivery systems for advances therapies and applications.

The interest in the study of marine polysaccharides for DDSs with therapeutic purposes relies in the possibility of developing novel approaches of less invasive and more personalized treatments. Several experiments have already shown that many of these biomaterials allow loading lower drug dosages, which may lead to a drastic reduction of the side effects caused by the drugs. These materials can be used as a signaling marker that could lead the delivery of a carrier to a specific location and widening the function of DDSs as diagnostic instruments [24,25]. These systems also have a wide applicability in gene therapy, which is usually limited by the health risk of associated with viral vectors [26]. In contrast, biomaterials have been shown to offer numerous advantages for the encapsulation of genetic material and others therapeutic agents, by ensuring stabilization and protection, also increasing its solubility and promoting a sustained release as well their biocompatibility and in some cases biodegradability [27,28]. In this review, we focus on the use of marine polysaccharides as raw materials for the construction of DDSs (Figure 1).

We identified alginate, chitosan, carrageenan, hyaluronan (also known as hyaluronic acid) and chondroitin sulfate as the major marine polysaccharides used currently in—or being considered for—the pharmaceutical industry. The various means to modify and adapt these biopolymers to achieve drug protection and delivery, stimuli-responsiveness and targeting capability will be discussed.

## 2. Polysaccharides from Marine Algae

**Figure 2:**
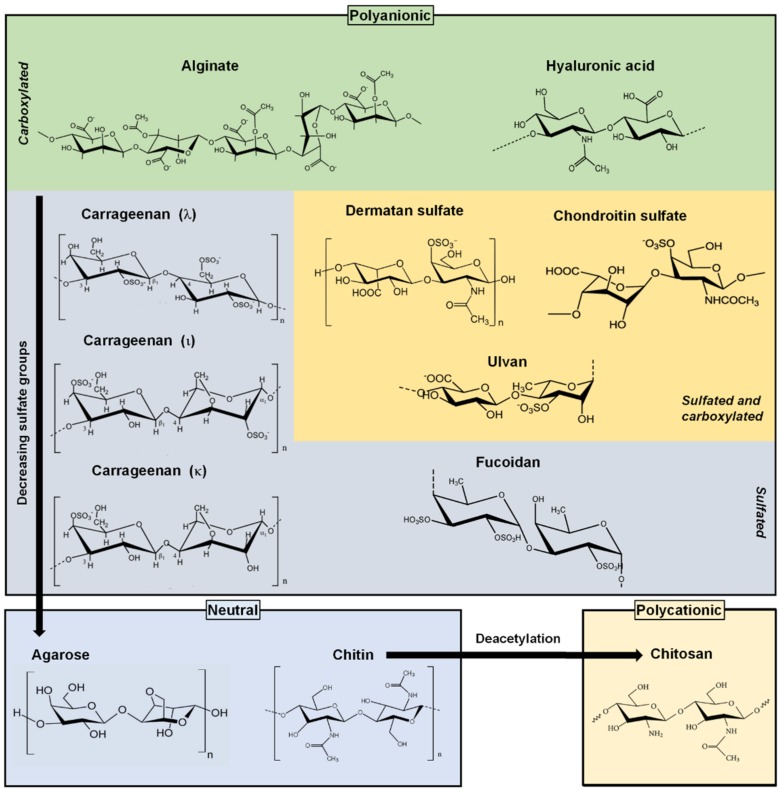
Marine origin polysaccharides categorized by electrostatic nature and carboxylated/sulfated structure.

Among the vast marine organism diversity, algae are the main source of marine polysaccharides. There are some polysaccharides that can be extracted from marine prokaryotes like microalgae, which can also be grown in bioreactors under controlled conditions. Red macroalgae are the most used sources of polysaccharides but it is possible to obtain polysaccharides from green or brown macroalgae. Seaweeds are a different type of multicellular marine algae and are also a major source of polysaccharides. The latter are also divided in groups: red, green and brown. Nowadays, the large quantity of marine algae that reach and deposit in coast regions has led to a widespread use of marine compounds to produce cosmetics, and food supplements and emulsifiers, among others. Despite their large bioavailability, polysaccharides remain relatively unexploited in the medical industry. Figure 2 represents the main polysaccharides that will be discussed herein.

### 2.1. Alginates

Alginate is a polysaccharide extracted from brown seaweeds, including *Laminaria hyperborea*, *Laminaria digitata*, *Laminaria japonica*, *Ascophyllum nodosum*, and *Macrocystis pyrifera* [29]. It is composed by a sequence of two (1→4)-linked α-l-guluronate (G) and β-d-mannuronate (M) monomers (Figure 2). The proportion of M and G blocks may vary with the type of seaweed that it is extracted from. For example, alginate extracted from *Laminaria digitata* and *Ascophyllum nodosum* have been shown to have M/G ratios of 1.16 and 1.82, respectively [30]. Alginate is biocompatible, has low toxicity and high bioavailability as well. These are the main advantages that make alginate one of the biopolymers with the widest biomedical applicability. One of the most common applications of alginate is their use as an excipient in DDSs, namely acting as a stabilizer agent in pharmaceutical formulations [31]. Alginate has carboxyl groups which are charged at pH values higher than 3–4, making alginate soluble at neutral and alkaline conditions [32]. Such pH sensitivity promotes greater protection for drugs with preferential absorption in the intestinal tract: the acidic environment of the stomach does not disturb the stability of the alginate carrier, whereas in the intestine (where the pH is alkaline) the solubility of this biopolymer—as well as the drug release—is promoted [33]. Thus, solubility and pH sensitivity make alginate a good biomaterial for the construction of DDSs [34].

Alginate is widely used for its biocompatibility, low toxicity, high bioavailability, lower extraction and purification costs as compared with other biopolymers, and for the capability to be processed in the form of hydrogel matrices, beads and particles [12,35,36,37]. Alginate is also used as an excipient in pharmaceutical tablets to promote greater protection and stabilization of the drug. Sodium alginate is the type of alginate mainly used in the pharmaceutical industry in the manufacture of tablets, especially when the drug is not soluble in water. Sodium alginate may be used for the purpose of extending the drug release [31]. Studies using tablets containing ibuprofen demonstrated that it is possible to control the absorption ratio of the tablets. By using sodium alginate with different chemical structure and degree of viscosity, Sirkia *et al.* obtained carriers that triggered either an immediate ibuprofen release or prolonged it, proving that the chemical structure of alginate may influence the release rate of the bioactive agent [38].

In acidic environments, alginate carboxyl groups are protonated, *i.e.*, in the –COOH form, being thus uncharged and exhibiting higher viscosity [32]. This may interfere with the elution of the bioactive agent from the device, thereby limiting drug release when the pH is low [39,40,41]. However, gelling sodium alginate with Ca^2+^ ions can solve pH dependent limitations related to the hydration, dilation and erosion of the carrier. Alginate has the ability of cross-linking with Ca^2+^ ions through an ionotropic gelation process, usually above pH 6 [42]. Ca^2+^ is not the only ion capable of promoting ionotropic gelation of alginate: Ba^2+^ or Zn^2+^ ions may also be used for that propose [43]. Virtually any drug may be entrapped during such mild cross-linking process, and its subsequent release may be dependent on several factors, such as cross-linking extension [44]. Giunchedi *et al.* reported that using sodium alginate, hydroxypropyl methylcellulose (HPMC), calcium gluconate, and ketoprofen as a model drug in the preparation of tablets by direct compression in different combination and ratios may prolong drug release, in particular in tablets with 20% of HPMC [45]. Alginate hydrogels also have applications in wound healing treatments through the construction of structures used for wound dressings. Several studies show that the bioavailability of drugs encapsulated in alginate hydrogels is greater than if free drug was applied directly at the lesion site, thus increasing the efficacy of healing [46]. Alginate hydrogels are also used widely in tissue regeneration treatments and cell encapsulation [47,48]. Hydrogels obtained from alginate, in particular, present some similar features of the extracellular matrix, thus being appropriate materials to be used in tissue engineering and regenerative medicine applications [46]. However, it should be noted that the gelling capability of alginate varies with the proportion of G and M groups, with alginates rich in G content yielding higher strength when compared to alginates rich in M groups [49].

**Figure 3:**
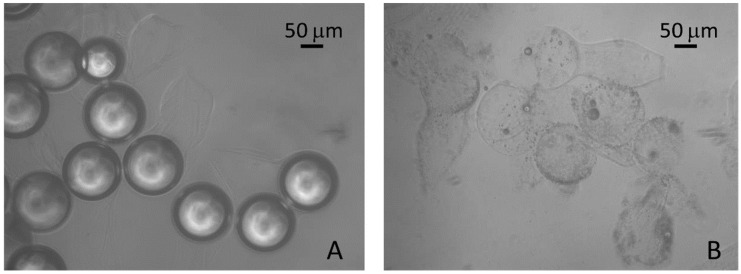
Optical microscope images of alginate microspheres before (**A**) and after (**B**) ultrasound exposure. Reprinted with permission from [51], Copyright © 2014 American Chemical Society.

Alginate is also used in the construction of microparticles with the ability to incorporate different bioactive agents, particularly proteins. Alginate microparticles have the capability of retaining large amounts of drug and also promoting protection of the cargo from any proteolytic attack. There are different mechanisms of release of a bioactive agent from the carrier, such as through variations of temperature and pH, and the use of biodegradable materials or enzymatic degradation, among other chemical and physical stimuli-responsive methods [32,33,35,50]. These parameters are difficult to control and program, since they can vary significantly. However, new release mechanisms from microparticles have been developed, that depended on fully controlled external stimuli, such as ultrasound-triggering. Duarte *et al.* developed a type of alginate microparticles which were shown to have perfluorocarbon breakthrough capacity when subjected to vibration by ultrasound waves [51]. Results showed a disruption of these microparticles after 15 min of exposure, suggesting that such structures are promising DDSs controlled externally by acoustic stimuli (Figure 3).

Over the years, other methods have been developed to fabricate drug delivery particles that promote a better loading efficacy of bioactive substances. Using superhydrophobic surfaces it is possible to produce polymer particles suitable as DDSs. This method allows loading drugs into spherical structures with an encapsulation efficiency close to 100% [52,53,54]. Another strategy to synthesize particles relies on complexation, based on the electrostatic interactions between alginate at neutral and alkaline pH values, bioactive agents and other kinds of naturally occurring polymers, such as the polycation chitosan [23,31,33,47]. In this matter, alginate complexes have been used to construct DDSs (especially nanoparticles) for gene therapy treatments. The very first systems for the gene delivery were based on genetic material encapsulated within viral vectors. These have several limitations such as the possibility to trigger an immune and inflammatory reactions, infections and mutations. These systems also have high costs of production due to complexity in the processing of viral vectors [26]. Taking advantage of the capability of natural polymers to form complexes with DNA, safer DDSs could be synthesized to deliver genetic material. The most commonly used polymers in the construction of DNA load vehicles are usually of synthetic origin, for example polyethylenimine (PEI), poly-l-lysine (PLL), poly(l-ornithine) and poly(4-hydroxy-l-proline ester) [55]. The use of these synthetic materials has allowed the synthesis of complexes via electrostatic interactions between the polymer and the DNA, allowing the creation of a stable complex and the possibility of size adjustment. One of the major limitations of using synthetic materials is their often adverse biological effect in the body. PEI, for example, exhibits elevated levels of cytotoxicity [56]. In contrast, most natural materials are biocompatibile, biodegradable (in some cases) and show similar capacity to form ionic bonds, therefore providing ensuring good protection for genetic material [57,58,59]. Krebs *et al.* developed a calcium phosphate-DNA nanoparticle system incorporated in alginate-based hydrogel for gene delivery to promote bone formation. Results showed a DNA sustained release from the alginate hydrogel around 45% of DNA released after approximately 75 days. *In vivo* studies, through the injection of alginate hydrogels containing calcium phosphate nanoparticles and osteoblast-like cells in mice, showed evidence of bone formation [60].

Taking its anionic nature, alginate can be assembled with polycations as structures other than particles using layer-by-layer (LbL). LbL is used to fabricate ultrathin nanostructured films in a multilayer fashion based on complementary interactions between building blocks, such as polyelectrolytes [61,62,63]. This technique may be useful as a biomimetic approach applied in deconstructing and reconstructing the physiological conditions found in native biological environments, such as the human body [64]. Polyelectrolyte freestanding films (*i.e.*, films with a few micrometers in thickness) have been shown to be suitable drug reservoirs of biomolecules, such as growth factors and antibiotics [65]. This type of films exhibit a good cell adhesion, possibility of cargo entrapment and fast release by variations of electrostatic interactions strength, and also promote a sustained release due to the slow film degradation [66,67,68,69,70]. Such multilayer systems can be also used as barriers with controlled mass transporter properties [71]. The versatility of LbL allows it to be extrapolated to the third dimension to conceive more complex DDSs, such as spherical capsules and tubular structures [72].

Microcapsules are also typical shapes of alginate processing following different techniques, including emulsion [73,74,75], multiple-phase emulsion [31,76] and calcium cross-linked encapsulation [77]. The ability of alginate to create complexes with other biomaterials by electrostatic interactions, chemical modification or cross-linking can be exploited for building hybrid and more versatile DDSs. Capsules constructed from chitosan/alginate-PEG complexes are reliable models for encapsulating proteins, such as albumin, one of the most common model proteins used in controlled release studies [78]. The construction of alginate spherical structures with other types of synthetic materials can be a good strategy to extend the versatility of these systems. Using poly(*N*-isopropylacrylamide) (PNIPAAm) to take advantage of its thermosensitive properties [79] in combination with alginate can lead to devices capable of delivering biomolecules with a dual stimuli-responsive dependence (both pH and temperature) [80]. Studies using indomethacin as a model drug reported that chitosan-alginate-PNIPAAm beads showed lower release rates with decreasing temperatures [81]. The same occurs when there is a decrease in pH, indicating that it is possible to control the permeability of the particles by controlling both pH and temperature. This approach can lead to the development of DDSs capable of promoting higher control over the release of drugs, proteins and others biomolecules with pharmaceutical interest. Following a similar concept of polymer conjugation, alginate can also undergo complexation with natural polymers, like chitosan, to enhance the absorption and cargo protection in oral delivery, for example, for the administration of insulin [73,82].

Alginate may be used in the construction of capsules for cell encapsulation often associated with cytotherapy treatments or simply the creation of cellular microcultures in more complex systems where the use of a conventional bioreactor is not possible. In this context, a new approach to the construction of alginate-based capsules for the incorporation of different types of cells has been presented [83,84]. Cells were encapsulated in alginate liquefied particles, coated with multilayer of alternating chitosan and alginate. Along with the cells, poly (lactic acid) microparticles were co-encapsulated to provide anchorage points so that cell survival is promoted. Results demonstrated a high viability of the encapsulated cells and usefulness of these capsules as culture systems. This type of system has wide applicability not only for the cell culture but also in other biomedical applications, since it will allow the encapsulation of different types of cells in combination with other biomolecules such as, for example, growth factors and other cytokines.

### 2.2. Carrageenans

Carrageenan is a sulfated polysaccharide present in red algae, which structure consists in a linear sequence of alternate residues forming (AB)*_n_* sequence, where A and B are units of galactose residues. These residues may or may not be sulfated. They are linked by alternating α-(1→3) (unit A) and β-(1→4) (unit B) glycosidic bonds (Figure 2). Unit A is always in d- conformation, while unit B can be found either in d- or l-configuration. The sulfated groups give it a negative charge, which categorizes carrageenans as polyanions [85]. Carrageenans are classified according to their degree of sulfation: they can be kappa (κ), iota (ι), and lambda (λ), if they have one, two or three sulfate groups respectively. The extraction process is straightforward, consisting in the immersion of the raw material in alkaline solution so that a gel forms. Then follows an extraction step, where the gel is immersed in water heated at 74 °C. Depending on the type of carrageenan and desired degree of purification, it is possible to execute additional purification steps, such as dialysis. The process finishes with filtration, precipitation and drying [85]. κ and ι types are most frequently extracted from algae of the *Kappaphycus* and *Eucheuma* genera, while λ type is often extracted from algae belonging to the family Gigantinaceae. The number of sulfated groups influences the gelation capability. Carrageenans κ and ι can form gels in the presence of cations, while the high sulfation degree of λ carrageenan prevent its gelation. Gelation capability has been used in many areas, such as food industries (using carrageenan as an emulsifier and stabilizer), as well as in the cosmetic and pharmaceutical industries [86].

Contrary to what happens with other biomaterials of marine origin, the use of carrageenan as an excipient in the pharmaceutical industry is not common, thus reports about their applications, characteristics and functions are infrequent. As an example, a study was conducted where two types of carrageenan (κ and ι) were analyzed in terms of compression behavior and their capability of tablet formation [87]. Results showed that both carrageenans are suitable excipients for controlled release. Carrageenans are also present in various biomedical applications due to their anticoagulant properties [88], antitumor, immunomodulatory [89], anti-hyperlipidemic [90] and antioxidant activities [91]. They also have a protective activity against bacteria, fungi and some viruses [92,93]. Due to the latter, carrageenans have been suggested for possible treatments of respiratory diseases, such as the famous bird flu, and is also being tested for killing other viruses, such as the dengue fever, hepatitis A, HIV [94] and herpes viruses [95]. Studies showed that carrageenan, and derivatives of degradation have different levels of toxicity, but do not endanger the health of the patients [93,96]. These properties make carrageenan a promising biomaterial for biomedical applications.

The use of carrageenan as an excipient in the manufacture of devices for oral delivery depends mostly on their physicochemical properties, such as water solubility and jellification capability. Carrageenan load capacity depends largely on the sulfation extent, which affects its mechanical properties and its dissolution rate. These factors may affect the release of the cargo, prolonging or accelerating its release [97]. A greater control over the drug release profile—regardless of other conditions, such as carrageenan type and pH—is possible by association or conjugation with other polymers. The addition of polymers such as hydroxypropyl methylcellulose (HPMC), a temperature sensitive semi-synthetic polymer, can solve problems related to pH erosion and provide higher protection to the drug, thus promoting a sustained release that does not depend on pH [98]. However, the opposite response may be desired (*i.e.*, pH-triggered degradation) and, for that, pH responsive polymers may be conjugated. By varying the pH, it is possible to control not only the loading but also the release mechanisms of carrageenan/alginate interpenetrated networks [99]. The use of stimuli-responsive materials offers another perspective for drug and gene delivery where the carrier may be an active trigger and function as a therapy optimizer. Using temperature-sensitive materials for nanocarriers construction can promote a controlled release at temperatures above 37 °C. Such a system could be helpful in situations as common as a fever. However, it is possible to use other nanocarriers in situations of hyperthermia, where the drug would be available in a localized region [100,101].

Carrageenan in the pharmaceutical industry is generally used as a raw material for the construction of DDSs, cell capsules for cell therapies and cartilage regeneration applications [27,102]. The use of carrageenan-based hydrogels as a vehicle for the controlled delivery of biomolecules can be a good strategy especially for cargo stabilization Popa *et al.* showed that κ-carrageenan hydrogels are adequate environments to encapsulate different types of human cells achieving chondrogenic differentiation [103]. This system proved to have potential for cartilage regeneration strategies, not only due to the referred differentiation but also because these hydrogels can be easily injectable *in situ* and may be used as reservoirs for growth factors [104]. Carrageenan-based hydrogels, along with other materials of marine origin, have also proved to be suitable good devices for cell encapsulation [105,106]. New methods on the production of spherical beads and fibrillar carrageenan/alginate based hydrogel have been developed. Fibrillar hydrogels obtained by wet spinning showed great potential for applications as a cell carrier for cell delivery systems [107]. Knowing the biological properties of carrageenan, it is hypothesized that carrageenan-based devices are suitable DDSs for the delivery of not only bioactive agents but also of cells for cytotherapies.

**Figure 4:**
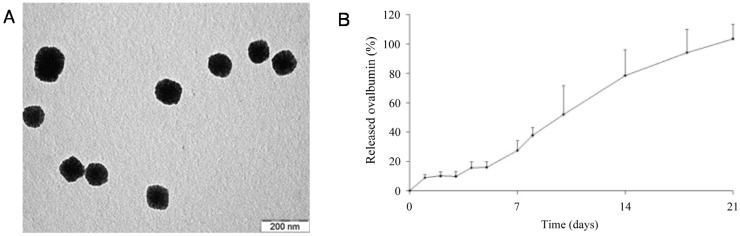
Transmission electron microscopy (TEM) micrograph of chitosan/carrageenan nanoparticles (**A**). Ovalbumin release profile from chitosan-carrageenan nanoparticles (**B**). Adapted with permission from [108], Copyright © 2009 Wiley Periodicals, Inc.

Taking advantage of the polyanionic nature of carragenans, they can be combined with polycations via electrostatic interactions. Grenha *et al.* developed carrageenan/chitosan nanoparticles through a simple construction method by ionic interactions between polycationic groups of chitosan and polyanionic ones of carrageenan (Figure 4A) [108]. This method has the advantage of avoiding the use of organic solvents and harmful cross-linkers. These nanoparticles had a diameter size between 350 and 650 nm. Using albumin as a model protein, *in vitro* release tests demonstrated a prolonged release over time, with a 100% of albumin release after three weeks (Figure 4B). Having a slow release rate is important since it enables the reduction of the encapsulated dose and also provides continuous long-term release without the need for repeated administrations. Cytotoxicity tests demonstrated that these devices present low toxicity. These results are a good indicator that these structures may be feasible for the encapsulation of agents with therapeutic purposes. Carrageenan has also been used in the construction of multilayer structures [109], microcapsules [110] and micro/nanoparticles [111].

### 2.3. Fucoidans

Fucoidan is a sulfated polysaccharide found in many species of brown algae. It is a polymer chain of (1→3)-linked α-l-fucopyranosyl residues (Figure 2), although it is possible to find alternating (1→3) and (1→4)-linked α-l-fucopyranosyl residues. The structure of fucoidan and its composition depend largely on the extraction source, especially the type of algae. For example, fucoidan extracted from *Fucus vesiculosus* is rich in fucose and sulfate, whereas that obtained from *Sargassum stenophyllum* contains many more types of residues besides fucose and sulfate, such as galactose, mannose, glucuronic acid, glucose and xylose. A more detailed comparison between several fucoidans and their extraction sources can be found elsewhere [112]. The extraction can be processed by precipitation using salts or organic solvents, followed by a purification step by chromatography. Recently it was reported that fucoidan has antitumor activity dependent on the degree of sulfation and can inhibit tumor cell proliferation and growth [113,114]. However, fucoidan may have inhibitory effects over some cellular functions. Cumashi *et al.* demonstrated that fucoidans may exhibit strong antithrombin properties and suppresses tubulogenesis on HUVECs [22]. Fucoidan has also shown anticoagulant and anti-inflammatory properties, as well as anti-adhesive and antiviral properties [115,116].

**Figure 5:**
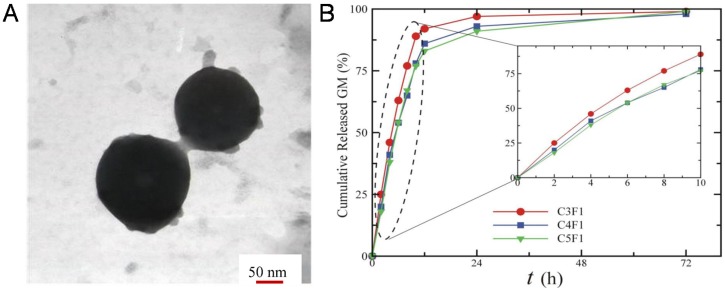
TEM image of chitosan/fucoidan nanoparticles (**A**). Gentamicin release kinetics from chitosan/fucoidan particles (**B**). Adapted with permission from [119], Copyright © 2014 distributed under a Creative Commons Attribution License.

Like other marine polysaccharides, fucoidan can also be used as a raw material for the construction of DDSs. A typical way of processing fucoidan DDSs is by electrostatic interactions with chitosan, to make microspheres, so-called fucospheres [117], which have been suggested for burn treatments. Particles with sizes ranging between 367 and 1017 nm were shown to trigger both *in vitro* and *in vivo* a decrease of the normal burn treatment time due to the increase of regeneration and healing of epithelial tissue [118]. Taking advantage of the great bioactivity of fucoidan, and the ability to complex with other materials like chitosan, other approaches can be pursued. Huang and Li developed novel chitosan/fucoidan nanoparticles with antioxidant properties for antibiotics delivery (Figure 5A) [119]. These nanoparticles presented a spherical morphology and diameter of 200–250 nm. Results showed a highly anti-oxidant effect by reducing concentration of reactive oxygen spices (ROS), using gentamicin as a model drug, release studies showed a controlled release around 99% of gentamicin in 72 h (Figure 5B). The antioxidant chitosan/fucoidan nanoparticles could thus be effective in delivering antibiotics to airway inflammatory diseases, where the amount of ROS it significantly high. Another approach to take advantage of chitosan/fucoidan interactions as DDSs is to synthesize hydrogels, as described by Nakamura *et al.* The authors developed a chitosan/fucoidan microcomplex hydrogel for the delivery of heparin binding growth factors, which showed high affinity with growth factors and were able to promote growth factor activity and also a controlled release [120]. *In vivo* studies showed a neovascularization promoted by the growth factors released from the chitosan/fucoidan hydrogel.

Another shape that can be obtained resorting to the polyanionic character of fucoidan are capsules, processed by LbL, particularly fucoidan-chitosan pH sensitive capsules for insulin controlled release [121]. Pinheiro *et al.* used polystyrene nanoparticles with a diameter approximately 100 nm as a template for the deposition of a fucoidan-chitosan multilayered coating [122]. After construction of the coating, the polystyrene core was removed, being thus possible to incorporate into the capsule numerous bioactive agents. Using PLL as a model molecule, results showed that the release profile was pH dependent and also that the release occurred by diffusion. These results indicate the sensitivity of these particles to pH variations found along the gastro-intestinal tract and the possibility of using these particles as DDSs for oral administration.

### 2.4. Ulvans

Ulvan is a sulfated polysaccharide extracted from the green algae of the *Ulva* and *Enteromorpha* genera. Ulvan consists in a polymer chain of different sugar residues like glucose, rhamnose, xylose, glucuronic and iduronic acid with α- and β-(1→4) linkages (Figure 2). Because of the large number of sugars in its composition, ulvan may exhibit variations in the electronic density and charge distribution, as well as variations of molecular weight. Since it contains rare sugars, ulvan is a natural source for obtaining them upon depolymerization, instead of resorting to chemical synthesis. The extraction process is simple, consisting in adding an organic solvent over the feedstock followed by successive washing steps with hot water, filtration and centrifugation [123]. Ulvan has several properties of biological interest, such as exhibiting antiviral, antioxidant, antitumor, anticoagulant, anti-hyperlipidemic and immune system enhancing activities. Ulvan also presents low cytotoxicity levels in a wide range of concentrations [124]. Ulvan is typically used in the food and cosmetic industries, but because of their biological properties, it has a great potential for the development of new DDSs, such as being used as an active principle in pharmacological formulations [125]. Because of their ability for complexing with metal ions, ulvan can also be used as a chelating agent in the treatment against heavy metal poisoning [126]. Furthermore, the capacity to process ulvan as nanofibers and membranes has been useful for tissue engineering and regenerative medicine, for example in wound healing treatments [127].

Ulvan has been used in construction of nanocarriers for biomolecules. Alves *et al.* constructed a two-dimensional ulvan-based structure for drug delivery by chemical cross-linking for wound healing [128]. Using dexamethasone as a model drug, there was a rapid release in the first hour (around 49%), followed by a slower and sustained release, around 75% up to 14 days. Additionally, it is also possible to obtain three-dimensional ulvan-based structures. In this context, ulvan/chitosan particles were produced for the encapsulation and release of dexamethasone [129]. These particles were incorporated in three-dimensional poly (d,l-lactic acid) porous scaffolds for bone tissue regeneration. *In vitro* release assays demonstrated a fast release in the first three hours (around 52%), followed by a sustained cumulative release up to 60% in the next 21 days.

Like other marine polysaccharides, ulvan may undergo chemical modifications to synthesize thermostable hydrogels. The addition of other functional groups is also possible so that temperature and light responsive hydrogels are conceived. In this case, ulvan was modified with methacrylate groups to allow jellification by photopolymerization through the irradiation with ultraviolet light [130]. This is a useful approach to develop cell encapsulation strategies for cytotherapy applications. Ulvan is also used in the construction of membranes, due to electrostatic interactions with other cationic polymers [131]. Through chemical modification, ulvan and chitosan can also be used as a polymeric component of bone cement, especially due to their mechanical properties [132].

## 3. Polysaccharides from Marine Animals

There are other marine sources beside algae and microorganisms: marine animals are also an excellent source for polysaccharides. In this section, the most important animal origin polymers used in DDSs will be presented. There are two main categories of polymers: chitin-derived polymers and glycosaminoglycans (GAGs).

### 3.1. Chitosans

Chitosan is a linear polysaccharide derived from chitin, one of the most abundant natural polymers of our ecosystem [133]. Chitosan is obtained by the deacetylation of chitin, resulting in a compound with randomly distributed d-glucosamine residues (deacetylated unit) and n-acetyl-d-glucosamine (acetylated unit) (Figure 2) [134,135]. Chitosan, as well as chitin, can be degraded by enzymes such as chitinase and lysozyme [136]. Chitin is the main component of the exoskeleton of arthropods and crustaceans such as crabs, shrimps and lobsters, and can also be extracted from some fungi and nematodes. Chitin is not water soluble, and thus it is usually converted into soluble derivatives including chitosan (soluble in acidic conditions) and carboxymethyl chitosan (soluble in a wide range of acidic and alkaline solutions). Chitosan has amine groups sensitive to pH variations, being positively charged in acidic environments and neutral in alkaline pH values (pK_a_ close to 6) [32]. Chitosan is one of the marine polysaccharides most widely used and studied for biomedical applications, in particular in the construction of nanoparticles, beads and capsules for controlled drug delivery systems, and also membranes, films and scaffolds for tissue engineering and regenerative medicine [137,138,139,140].

Chitosan has antimicrobial activity, a useful property to build films that prevent wound infection [141,142]. It also shows antitumor and anti-inflammatory activity [143,144]. All of these biological properties make chitosan an excellent candidate for constructing devices that require the contact with biological environments, and as excipients for DDSs [145,146]. For chitosan-based DDSs, electrostatic interactions between the polysaccharide and a bioactive agent are a key to drug stabilization, protection and acceleration (or deceleration) of its release. This means that, if a drug is anionic, positively-charged polymers (like chitosan) are used as excipient, and *vice-versa*. The release profile and rate of biomolecules from within chitosan-based carriers may depend on the morphology, size, density, cross-linking degree, as well as the deacetylation degree of chitosan and physicochemical properties of the bioactive agent. The release will also be affected by the pH and by the presence or absence of enzymes. The release may occur in different ways: (i) release from the surface of DDSs; (ii) passive diffusion; and (iii) erosion of the DDS. Deacetylation degree of chitosan can be also used as a degradation control parameter [137,147]. Another mechanism of release exploited for chitosan-based carriers is triggered by enzymatic degradation [148].

It is also possible to increase the binding capacity of poorly water-soluble drugs by introducing different chemical modifications onto chitosan. Chitosan chemical modification can be a good strategy to increase the effectiveness of release and attribute other properties such as drug protection and stabilization [135]. Hydroxypropyl chitosan (HPCH), obtained from the reaction between chitosan and propylene epoxide under alkali condition, can be grafted with carboxymethyl β-cyclodextrin mediated via a water-soluble 1-ethyl-3-(3-dimethylaminopropyl) carbodiimide (EDC) [80]. Hydrophobic drugs can be encapsulated due the presence of hydrophobic groups present in HPCH. In addition, due to the free amine groups that can be protonated at lower pH values such DDSs can be pH-responsive. Using ketoprofen as a hydrophobic drug model, *in vitro* release results showed that this chitosan derivate has a great potential as a biodegradable delivery system for hydrophobic drugs in a pH-sensitive controlled release [149,150]. The introduction of thiol groups has also been shown to increase the solubility of chitosan in water, maintaining the pH dependence of chitosan particles [151]. *N*,*O*-carboxymethyl chitosan (NOCC, also known as carboxymethylated chitosan) is a water soluble derivative that retains a fraction of the amine residues and its polycationic properties under acidic conditions [34]. Ketoprofen-loaded beads of NOCC and a PNIPAAm with a telechelic amine group (PNIPAAm-NH_2_) were developed for the study of controlled release system. Release studies taking in acidic and physiological conditions at 21 and 37 °C showed that these particular beads are sensitive to temperature and pH variations [152]. Acetylated chitosan grafted with fatty acid like palmitoyl is another strategy to develop chitosan-based excipients to entrap and release hydrophobic drugs [153,154,155]. Photo-sensitive products can also be synthesized. Methacrylamide chitosan, a water-soluble modified chitosan, is suitable for photo-cross-linking and has been used for the construction of delivery carriers. Wijekoon *et al.* developed a fluorinated methacrylamide chitosan hydrogel for oxygen delivery in wound healing [156]. During the methacrylation process, different fluorinated ligands were added to chitosan to obtain different fluorinated methacrylamide chitosans. Hydrogels were constructed by photo-cross-linking. This new biocompatible, injectable moldable photo-cross-linked chitosan-based hydrogel allowed controlling both the capacity and rate of oxygen delivery, maintaining beneficial oxygen level up to five days.

The reactivity of chitosan with other materials may also promote sustained release, as well as cargo stabilization and protection. This can be achieved using different methods, such as graft copolymerization with synthetic polymers like poly(ethylene glycol) (PEG) and PEI [148,157]. Several studies showed the ability of chitosan to enhance and prolong the absorption of hydrophilic drugs taken by oral [158] and pulmonary [159] administration routes. Chemical modification of chitosan with PEG is a way of improving the biocompatibility of chitosan, especially to reduce chitosan toxicity, as well as to enhance protein adsorption, cell adhesion, growth and proliferation [160,161]. Prego *et al.* showed that chitosan-PEG nanocapsules for oral delivery of peptides exhibited low cytotoxicity and enhanced intestinal absorption capability [162]. Other studies showed that this approach can also be applied to deliver other drugs such as insulin [163,164,165,166]. Taking advantage of the jellification capability of some copolymers containing chitosan, Bhattarai and coworkers presented an injectable PEG-grafted chitosan hydrogel for controlled release [167]. These hydrogels were liquid at room temperature and a gel at physiological temperature. Using albumin as a protein model, *in vitro* release studies at 37 °C showed a high release in the first 5 h, up to 50%–60% followed by a sustained release for the next days with a cumulative release up to 80%.

Hydrogels based on cross-linked chitosan may have the ability to promote a sustained release upon nasal administration. Hydrogels were constructed by joining *N*-[(2-hydroxy-3-trimethylammonium) propyl] chitosan chloride (HTCC) and PEG with the addition of a small quantity of α-β-glycerophosphate (α-β-GP) as a gelling agent [168]. These hydrogels are pH sensitive and have the particularity of being liquid at room temperature and exhibit higher rigidity at 37 °C. Wu and coworkers developed these hydrogels as smart devices for the controlled release of biomolecules through nasal administration as drops or spray. Once applied, the solution is exposed to physiological temperature, becoming a viscous hydrogel which can be absorbed by mucosa. Because of their ease of production and administration, this new formulation was tested as a loading device for the controlled release of insulin. Assays in rats showed an increased absorption in the nasal cavities and a decrease in blood glucose, without any evidence of cytotoxicity. These results demonstrated the great potential of these hydrogels as carriers for the controlled release of bioactive agents, especially hydrophilic biomolecules [169]. Nasal administration is less compliant for the patient, causing no discomfort and pain, leading to a reliable management and patient satisfaction [170]. Furthermore, the fact that this type of hydrogels are liquid at room temperature also enhances their ease of application as a DDS for parenteral administration [171].

Chitosan can form stable and highly dense electrostatic complexes capable of providing stability and protection to drugs. Being a polycationic polysaccharide, chitosan can form particle complexes with nucleic acids for gene therapy [172,173,174,175]. The formation of particle complexes between the polymer and the nucleic acids depends on many intrinsic factors, such as the deacetylation degree, the molecular weight, as well external factors like temperature and pH, and represent crucial factors on the efficiency of transfection [176]. The positive charge of chitosan allows interacting with the negatively charged peptidoglycans present in the cell membrane, facilitating the entry of a chitosan/DNA complex into the cell by pre-established endocytic pathways [177,178]. The amount of genetic material available to react with chitosan is also very important: an improper ratio can lead to the dissociation of the complex or to a lack of synthesized complexed particles, resulting in low transfection rate [176]. It has been reported that using chitosan overcomes some of these limitations: chemically modifying chitosan can increase the affinity with the DNA to yield a more stable complex, which can lead to an increase in the transfection efficacy [179]. The modifications can also increase chitosan solubility and thus offer greater protection to the cargo from the degradative action of DNases on DNA [176]. Chemical derivatives opened a new range of possibilities to construct DDSs for the intracellular release of the genetic material, with wide applicability in the treatment of various genetic diseases. Following this line, Forrest and coworkers presented a PEI-PEG-chitosan-copolymer for gene delivery with good loading capacity and high transfection efficacy, as well as low toxicity that makes these particles good candidates for *in vivo* gene delivery [180].

Chitosan-based capsules can also be synthesized resorting to electrostatic complexation, and, in some cases, are able to respond to external stimuli other than pH. One such example is the conception of LbL microcapsules made by complexation of chitosan with negatively charged elastin-like recombinamers (ELRs), recombinant polypeptides with intrinsic response towards temperature [181]. Novel thermoresponsive ELR/chitosan microcapsules were developed for the delivery of active molecules [182]. Using bovine serum albumin (BSA) as a model molecule, the results showed a greater BSA retention at physiological temperature (37 °C), when compared to room temperature (25 °C). Studies with cells also showed a low cytotoxicity for such structures. The pH response of these microcapsules was not studied, but the results are a good indicator that chitosan can bond with other sources of stimuli-responsive biomaterials, including unconventional ones such as genetically engineered polypeptides. While thermal responses are perhaps the most exploited mechanism integrated in smart DDSs, it is debatable whether their sensitivity would be enough to treat, for example, a common fever, where the body temperature varies just 1–2 °C. Besides, not all people have exactly the same body temperature. Therefore, conjugating two or more physiological parameters could be a solution for diseases that require administration based on triggers operating within tight ranges.

### 3.2. Hyaluronans

Hyaluronan belongs to the family of glycosaminoglycans. GAGs are linear, negatively charged heteropolysaccharides composed of repeating disaccharide units of N-acetylated hexosamine and uronic acid (with the exception of keratan sulfate) [183]. It is a linear polysaccharide consisting of an alternating chain disaccharide units of *n*-acetyl-d-glucosamine and d-glucuronic linked by β-(1→3) and β-(1→4) glycosidic bonds (Figure 2) [184]. Hyaluronan is a major component of extracellular matrix and is present in the synovial fluid, vitreous humor and cartilage tissue. Due to its high viscoelasticity, hyaluronan has an important role in several biological functions and also as an excellent material for different biomedical applications. Namely, it is involved in tissue regeneration, cell proliferation, differentiation and migration [185]. Because of its presence in the synovial fluid in joints, hyaluronan can be used as a biological marker to diagnose diseases associated with rheumatoid arthritis [186]. Due to its biocompatibility and biodegradability, hyaluronan has also been proposed for tissue engineering applications for manufacturing wound healing structures [187] and as a supplement for patients with arthritis [185]. Nowadays, hyaluronan production is done on a large scale using different methods and sources, such as bacterial fermentation [188,189,190]. hyaluronan may also be extracted directly from marine animal sources, such as cartilage and also from the vitreous humor of several fish species [191]. His biodegradability is mediated by the action of hydrolases, such as hyaluronidase, which breaks the glycosidic bond between two residues [192]. In the human body, hyaluronan is present in various biological fluids, allowing its use as a biomarker to monitoring its movement in biological fluids [193,194].

Hyaluronan hydrogels with dual stimuli-responsiveness can be made, namely towards pH and temperature variations. Hydrogels were obtained from hyaluronan and PNIPAAm with TEMED as a cross-linker [195]. Using gentamicin as a model drug, *in vitro* release assays at 37 °C and pH 7.4 showed an initial release of around 25% in the first 60 min, followed by a sustainable release up to 30% over the following 20 h. These results also showed that the release rate increases with increasing hyaluronan ratio in the hydrogel composition. These structures showed sensitivity to variations in temperature, showing potential as a device for biomolecules loading with smart controlled release system. There are other interesting types of hyaluronan conjugate-based hydrogels. Hyaluronan-tyramine (HA-Tyr) conjugates can be obtained by the enzymatic oxidative reaction of tyramine moieties using H_2_O_2_ and horseradish peroxidase (HRP). These hydrogels are highly biodegradable, which can be controlled by the cross-linking degree [196], and can encapsulate drugs. It was reported that the concentration of H_2_O_2_ has an influence in the mechanical strength of the hydrogel and on the release rate of drugs [197]. It was also reported that, in contact with hyaluronidase, the entrapped protein can be released continuously and completely from a hydrogel due to the polymer network degradation. On the same line of work, a new hyaluronidase incorporated-hyaluronan-tyramine hydrogel was developed for the delivery of trastuzumab, an antibody drug against breast cancer. *In vitro* release studies showed an antibody tunable release accompanied by the hydrogel degradation controlled by the concentration of hyaluronidase, as well as trastuzumab-dependent inhibition on the proliferation on cells [198].

Like other polyanions, hyaluronan can be complexed with polycations such as chitosan to form nanoparticles [199] and microspheres [200]. Recent studies presented these systems as a new approach for the treatment of ocular disorders. Hyaluronan/chitosan nanoparticles have been synthesized by means of electrostatic interactions to develop nanoparticles for the delivery of genes to the cornea and conjunctiva [201,202]. Results indicated an appropriate size distribution (100–230 nm) and internalization of these particles by endocytic processes mediated by membrane receptors. This result reveals the great biomedical applications potential of these nanoparticles as gene delivery device for treating diseases at the level of the human conjunctiva and other ocular diseases.

Hyaluronan has also been used as a coating material for spherical structures. Cross-linked chitosan spheres can serve as templates for the alternating adsorption of hyaluronan and chitosan multilayers [203]. *In vitro* release using gentamycin sulfate as a model drug indicated a sustained release from the microspheres, compared to the release from uncoated cores. These results show that a LbL coating can promote stabilization to the cargo and for that reason allows an enhanced sustained release. Liposomes are also viable spherical templates for hyaluronan coatings. Liposomes are pH sensitive lipid-based structures, and have been used as carriers for the controlled release of bioactive agents for cancer treatments [204]. One useful application of such pH sensitiveness is for the intracellular delivery of peptides. Jiang *et al.* presented a new liposome coated with hyaluronan for anticancer drug delivery [205]. In this case, the coating protected the liposome and the cargo against attacks by proteins present in the bloodstream. Entering the tumor extracellular matrix, where the hyaluronidase degrades the outer layer of hyaluronan, exposes the liposome to pH changes existing in the cytoplasm, enabling the intracellular drug release. A high antitumor activity was also detected during *in vivo* tests.

One interesting feature of hyaluronan is the ability to interact with several proteins. Such capability can be useful as a diagnostic tool, in particular due to existence of membrane receptors specific for hyaluronan. It is the case of CD44, a receptor that is highly expressed when there is an increase in cell proliferation. Determining an increased expression level of CD44 by means of hyaluronan devices can be an excellent marker for the early diagnosis of cancer [206]. Hyaluronan hydrogels can be used as reservoirs of bioactive agents obtained via various methods of constructions [207]. Nanoparticles based on the interaction of hyaluronan with metals, such as gold, have been widely used as markers for diagnosing diseases such as rheumatoid arthritis and cancer due to the ability of some of these devices to emit fluorescence [194,208,209,210].

### 3.3. Chondroitin Sulfates

Chondroitin sulfate is a sulfated glycosaminoglycan composed of a single chain of repeating disaccharide units of glucuronic acid (GlcA) and *N*-acetylgalactosamine (GalNAc) linked by β-(1→3) and can be sulfated in different carbon positions (Figure 2). It is usually extracted from the cartilage of bovine and porcine cattle but can also be extracted from some marine animals, like the whale and shark. However, due to ecological reasons, the extraction of protected species is currently quite limited. There are nonetheless other non-mammalian marine animal sources, such as the ray, the salmon fish, the sea cucumber, some cnidarians and mollusks [27]. Chondroitin sulfate has anticoagulant properties and has been suggested as a natural substitute for heparin, one of the most widely used anticoagulants [211,212]. In the pharmaceutical industry, this polysaccharide has been used as an active principle in drugs with anticoagulant properties, as a supplement to prevent arthritis [213], and as hydrogels for cartilage tissue regeneration [214]. Therefore, chondroitin sulfate is a suitable material to build DDSs. Studies with chondroitin sulfate/chitosan nanoparticles have indicated a large retention capacity of proteins and polypeptides, like growth factors [215]. Release assays showed a sustained release of the cargo in the order of 65% in the first 30 days. Studies *in vitro* performed on human adipose derived stem cells stem showed the ability of these nanoparticles to enter the cells promoting osteogenic differentiation. Cell internalization proved to be dependent on the particles concentration in the culture media, as well as on the incubation time.

Electrostatic interactions between different materials can be used for the construction of DDSs with the ability to incorporate different bioactive agents, as well as to enhance the cargo loading and to promote a sustained controlled release [216]. Despite the numerous advantages of using natural materials, synthetic polymers are still commonly used in the pharmaceutical industry, though they can be conjugated with natural ones. For example, chondroitin sulfate/PEG hydrogels was developed and proposed for a variety of biomedical applications, such as in wound healing and regenerative medicine [217]. This type of hydrogels proved to be biocompatible, since no inflammatory response when implanted has been observed, and is also biodegradable by enzymatic activity.

While 3D hydrogels and spherical objects are common designs for DDSs, a recent study showed that porous tubular structures can be constructed from hydroxyapatite and chondroitin sulfate for the delivery of chemotherapeutics [218]. Results for doxorubicin hydrochloride release showed a high encapsulation capacity around of 91% of efficacy due to the tubes geometry and porosity. *In vitro* release assays at different pH values (5, 6, and 7.4) revealed a pH dependent controlled release. These results revealed the potential use of these structures as controlled drug delivery devices for chemotherapy treatments, not only because of their pH dependent release, but also due to the long-term sustained release that eliminates the need for regular administration.

## 4. Emerging Glycosaminoglycan-Like Polysaccharides from Marine Origin

There are several types of glycosaminoglycans with different biological properties but, unlike hyaluronan and chondroitin sulfate, their bioavailability is low, they are difficult to extract and to produce, therefore they are not widely used in pharmaceutical sciences. However, due to their biological properties, they can be used as active agents in supplements. Examples include the sulfated glycosaminoglycans dermatan sulfate, heparan sulfate and keratan sulfate, and the nonsulfated agarose.

### 4.1. Dermatan Sulfates

Dermatan sulfate is a glycosaminoglycan with a linear disaccharide chain containing units of hexosamine, *N*-acetyl-galactosamine or glucuronic acid linked by β-(1→4) or (1→3). In some cases, this compound may present residues of l-iduronic acid, being the main structural difference between dermatan sulfate and chondroitin sulfate. Dermatan sulfate is extracted mainly from ray skin and can be used as a stabilizer for growth factors and cytokines. Recent studies have shown anticoagulant activity for dermatan sulfate without causing the possible complications present in the treatments made with heparin [219,220,221]. Dermatan sulfate anticoagulant character inhibits thrombin, showing no effect on factor *X* of the clotting cascade. It also has no interaction in platelet function. Thus, dermatan sulfate is a good alternative for heparin [222]. Thanks to its anticoagulant and antithrombotic activities, dermatan sulfate is seen as a potential substitute for heparin [211].

### 4.2. Heparan Sulfates

Heparan sulfate is another glycosaminoglycan which structure is very similar to heparin. It consists in a linear chain of alternating d-glucuronic acid or iduronic acid and d-glucosamine residues, which can be sulfated or acetylated. The distribution of sulfated residues can set some of the biological properties of heparan sulfate. The number of sulfated groups can influence the affinity with other proteins and so may influence their biological properties [223]. For example, heparan sulfate can block DNA topoisomerase activity in cell nucleus [224], and also has a role in the control of cell cycle and proliferation. Regarding the latter, heparin sulfate/cell complexes are often associated with increased cell proliferation which can lead to processes of oncogenesis. Thus, heparan sulfate has a significant role in the development of cancer, which is being associated with the increase of cell proliferation, angiogenesis in tumors, cancer cells differentiation and metastasis formation [225]. However, the effect of heparan sulfate on tumor cells may depend on the glycosaminoglycan structure, the type of tumor cell and/or the tumor microenvironment [226].

Independently of its role in cancer, this sulfated polysaccharide is also biodegradable, particularly by enzymatic action of heparanase [227]. Due to the presence of sulfated groups, it may bind to a number of different proteins and regulate biological processes such as coagulation and regulation. Heparan sulfate has the ability to bind to various polypeptides, such as the complex formed by the cellular receptor and growth factors [228]. Chemical modification of heparan sulfate can interfere with its anticoagulant activity and can have therapeutic effects in tumors. Regardless of the heavy involvement of heparan sulfate in different stages of tumor formation, it is possible that this polymer could be helpful as a new diagnostic method in the discovery and in the development of new drugs for cancer treatments, as well as in the development of DDSs with sensing capability. [229]. Due to the biological properties of heparan sulfate, it is not unreasonable to state that the production of heparan sulfate-based DDSs based could be a strategic approach to develop new chemotherapeutic strategies.

### 4.3. Keratan Sulfates

Keratan sulfate is a glycosaminoglycan that lacks the uronic acid unit. The disaccharide unit normally consists in galactose residues and *N-*acetylglucosamine bonded by β-(1→4) linkages. The extremities of keratan sulfate have a protein binding region at the extremities. There are three different classes of keratan sulfate which differ in the nature of the protein binding region. Class I is known for its presence in the cornea and in small cartilage. The protein binding occurs between the *N-* of a *N-*acetylglucosamine and an asparagine. In Class II, also present in small cartilage, the protein binding is made between the *O-* of *N-*acetylglucosamine with either a serine or a threonine. Finally, in Class III (first isolated from nervous tissue), the protein binding occurs in the *O-* of the mannose residue to a serine or threonine [230]. The presence of keratan sulfate in corneal tissue is related to the maintenance of the moisture level of the corneal tissue, which may influence its levels of transparency. Studies at the cellular level have shown that keratan sulfate has anti-adhesive properties. In nervous tissues, keratan sulfate can prevent the growth of axons, and in cartilage it may decrease the immune response in diseases such as osteoarthritis [231]. However, keratan sulfate presents an inhibitory action in nerve regeneration after nerve injury [232,233].

### 4.4. Agarose

Agarose is a marine biomaterial with a structure similar to carrageenan, present in the cell wall of red algae. Its structure comprises monosaccharide residues connected alternately in the conformation (AB)*_n_*. The units consist of galactose residues linked by α-(1→3) (unit A) and β-(1→4) (unit B) linkages. The main difference between carrageenan and agar is that the carrageenan unit A is always in the d- conformation, while in the agar unit A can only be in the l- conformation [234]. Unlike carrageenan, agarose is not classified according to the sulfation degree, since the best known type of agarose is a neutral type without any sulfated group. Agarose is widely used in food industry and also in microbiology in the form of gel to be used as culture medium in the form of agar. Agarose is associated with several biomedical applications especially as hydrogels for the release of bioactive agents, taking advantage of its ability to jellify, biocompatibility and native biodegradability [235,236].

## 5. Conclusions

Marine polysaccharides have been widely used to synthesize DDSs. The fact that they are biocompatible, nontoxic and often biodegradable and stimuli-responsive makes these polymers suitable raw materials for the construction of increasingly complex loading devices with a release that can be potentially controlled. We showed that such devices can be constructed using different methods and can be synthesized in various shapes, such as hydrogels, particles and capsules, capable of protecting different bioactive agents like proteins and nuclei acids. Each and every polymer exhibits several chemical and biological properties, making marine biomaterials and their derivatives excellent materials not only for the construction of load devices but also for other pharmaceutical formulations as excipients or even active compounds in some food supplements. Natural-origin biomaterials allow incorporating a wide variety of proteins, drugs and nucleic acids, which for many new drugs would not be possible with many synthetic materials, which may be even toxic for the body. The release of bioactive agents may occur through various mechanisms, which may be controlled by using stimuli-responsive polymers to promote a fast or a sustained release. Because these materials are often biocompatible and biodegradable, their use may augment the efficiency of encapsulation and promote the protection of a bioactive agent.

Nowadays, it is already possible to find systems able to control the release of therapeutic molecules for the treatment of genetic diseases. Despite the great knowledge and wide use of marine polysaccharides in the pharmaceutical industry, some challenges remain unsolved, such as the efficient targeted delivery, the perfect control over the release rate to fit within a therapeutic window, and the adaptability to administration routes that are more patient compliant (e.g., oral instead of intravenous). Therefore, further investigation will be required to improve the isolation and purification of marine biopolymers, as well as the synthesis of their chemical modification and processing into the various possible matrices shapes. It is expected that in the short-term such control will lead to more efficient loading, higher degrees of control over the release and improved DDS designs, that could be used in advanced therapies. This could be possible by looking into the interactions between polymer, drugs and native biological tissues, as well the intelligent response of the polysaccharides and targeting capability. Future strategies should also combine the possibility of controlled release from this type of devices with diagnostic capability (theranostics approaches) where platforms involving nanotechnologies and image should be taken into consideration.
